# Unraveling the genomic secrets of *Tritonibacter mobilis* AK171: a plant growth-promoting bacterium isolated from *Avicennia marina*

**DOI:** 10.1186/s12864-024-10555-0

**Published:** 2024-07-05

**Authors:** Amal Khalaf Alghamdi, Sabiha Parween, Heribert Hirt, Maged M. Saad

**Affiliations:** 1 DARWIN21, Biological and Environmental Science and Engineering Division (BESE), King Abdullah University of Science and Technology (KAUST), Thuwal, 23955-6900 Saudi Arabia; 2 Department of Botany and Microbiology, College of Science, King Saud University, P.O. Box 2455, Riyadh, 11451 Saudi Arabia; 3 Max Perutz Laboratories, University of Vienna, Vienna, Austria

**Keywords:** Mangrove, Salinity, Red sea, Biocontrol, *Arabidopsis thaliana*, *Metabolites*

## Abstract

The scarcity of freshwater resources resulting in a significant yield loss presents a pressing challenge in agriculture. To address this issue, utilizing abundantly available saline water could offer a smart solution. In this study, we demonstrate that the genome sequence rhizosphere bacterium *Tritonibacter mobilis* AK171, a halophilic marine bacterium recognized for its ability to thrive in saline and waterlogged environments, isolated from mangroves, has the remarkable ability to enable plant growth using saline irrigation. AK171 is characterized as rod-shaped cells, displays agile movement in free-living conditions, and adopts a rosette arrangement in static media. Moreover, The qualitative evaluation of PGP traits showed that AK171 could produce siderophores and IAA but could not solubilize phosphate nor produce hydrolytic enzymes it exhibits a remarkable tolerance to high temperatures and salinity. In this study, we conducted a comprehensive genome sequence analysis of *T. mobilis* AK171 to unravel the genetic mechanisms underlying its plant growth-promoting abilities in such challenging conditions. Our analysis revealed diverse genes and pathways involved in the bacterium’s adaptation to salinity and waterlogging stress. Notably, *T. mobilis* AK171 exhibited a high level of tolerance to salinity and waterlogging through the activation of stress-responsive genes and the production of specific enzymes and metabolites. Additionally, we identified genes associated with biofilm formation, indicating its potential role in establishing symbiotic relationships with host plants. Furthermore, our analysis unveiled the presence of genes responsible for synthesizing antimicrobial compounds, including tropodithietic acid (TDA), which can effectively control phytopathogens. This genomic insight into *T. mobilis* AK171 provides valuable information for understanding the molecular basis of plant-microbial interactions in saline and waterlogged environments. It offers potential applications for sustainable agriculture in challenging conditions.

**Supplementary Information:**

The online version contains supplementary material available at 10.1186/s12864-024-10555-0.

## Background

Several methods have been attempted to address agricultural salinity challenges, such as developing salt-tolerant crops, soil amendments, crop rotation, and conventional breeding. Unfortunately, these approaches have proven inadequate, labor-intensive, and time-consuming. Consequently, it is imperative to implement alternative technologies to bolster agricultural sustainability, including using plant growth-promoting bacteria (PGPB) [1] with special emphasis on root microbiota. The root-associated biome comprises diverse microbial communities and blueprints the natural relationship between plants and microbes [2, 3]. Recently, several studies have demonstrated the efficacy of PGPB as modulators of salinity in plants and promoters of plant growth [4, 5]. Several mechanisms have been identified as contributing to stress mitigation, including biofilm formation, extracellular polymeric substances (EPS) production, nitrogen fixation, phytohormone production, and modulation of several plant’s hormonal pathways [6–8]. PGPB might induce the biosynthesis of several osmoprotectants, like trehalose, proline, glycine, phenols, and flavonoids, that reduce the reactive oxygen species (ROS) and reactive nitrogen species (RNS) in cells[Gupta, 2022 #2511][Kurniawan, 2021 #2513]. In addition, terpene-producing PGPR may support crop plant growth and yield [Oleńska, 2020 #2517]. Such as Bacillus and Pseudomonas, which increase the yield of apples, tomatoes, and peppers [Katsenios, 2021 #2518]. The regulation of terpene biosynthesis may enhanve the tolerance of cotton submergence under soil flooding [Sun, 2023 #2516].

The genomes of salt-tolerant bacteria and archaea found in marine environments offer a wealth of information about their unique adaptations to survive in extreme conditions. Several genomes of salt-tolerant bacterial and archaeal strains of the marine environment have been completed for over 44 taxa, including Alphaproteobacteria such as *Marinobacter* and *Roseobacter* [9], revealing a biodegradable potential in high salinity environments. The *Roseobacter* group is adapted to the marine environment [10] and comprises chemotrophic bacteria often associated with eukaryotes [11]. Members of this group have a much higher metabolic and ecological versatility than other dominant marine bacteria [12]. Producing compatible solutes such as choline, ectoine, and hydroxyectoine also enhances the bacteria’s ability to tolerate stresses such as salinity or high temperature [Vargas, 2008 #2519].

*Tritonibacter mobilis* is an alpha-proteobacterium from the abundant *Roseobacter* clade [13]. It is formerly known as *Ruegeria mobilis* [14], and *R. pelagia* [15, 16], representing 0.2% of the bacterial population in the surface ocean [17]. Nevertheless, *R. mobilis* exhibits extensive genome divergence and evolutionary events leading to the speciation of marine bacteria, where horizontally transferred genes mostly originate from bacteria of the *Roseobacter* group. In addition, *R. mobilis* has been shown to produce the antibiotic tropodithietic acid (TDA) as a multifunctional secondary metabolite. TDA is a weak iron-chelating [18] agent with hormetic effects on nearby organisms, such as antibiosis [19]. This bacterium is adapted to a surface-attached lifestyle [20], as one-third of TDA-producing bacteria are host-associated [21].

The marine environment is home to diverse microorganisms, many of which have evolved unique adaptations to survive in highly saline conditions. Salt-tolerant bacteria and archaea are particularly interesting to scientists studying extremophiles and their potential for industrial and biotechnological applications. Here, we present the complete genome sequence of the PGPB *Tritonibacter mobilis* AK171, isolated from the rhizosphere of mangroves. AK171 has salinity stress tolerance growth-promoting abilities on *Arabidopsis thaliana*. It provides new tools for enhancing our understanding of PGPR and applications in sustainable agriculture systems and a gold mine for several metabolites with potential industrial applications.

## Materials and methods

### The isolation and growth conditions of AK171

AK171 was isolated from the rhizoplane of the intertidal plant *A. marina* growing in the coastal area of the Red Sea (GPS: 22.339914°N, 39.087972°E), Thuwal, Saudi Arabia, as described previously [22]. In brief, the roots of *A. marina* were collected, and the excess soil was manually removed by shaking the roots and then placed in a sterile 0.9% phosphate-buffered saline (PBS) solution and vortex for 15 s. The cleaned root was placed sonicated for 30 s at 50–60 Hz. The liquid PBS “rhizoplane” compartment was serially diluted, plated on the filtered seawater yeast agar (FSY) supplemented with Luria-Bertani 35 gm/L (L7025, Sigma-Aldrich) and solidified with 2% Agar (SLB), and incubated at 30 °C until colony appears. AK171 was picked among > 300 isolates; based on initial identification and biochemical screening, AK171 was selected for further analysis. The colonies were cream-colored, opaque, circular, and convex with entire edges. The pure culture of AK171 was then regularly growing on/in Zobell Marine 2216E (ZM) (Bio Basic Asia Pacific Ltd, Singapore) at 30 °C.

### Biochemical assays

Plant growth-promoting (PGP) traits were evaluated by using clearing assays. The ability of AK171 to solubilize phosphate was assessed on Pikovskaya’s (PVK) agar plates (M520, Himedia). Using Blue Chrome azurol S (CAS) Agar assay, siderophore production was determined as described by Louden et al. [23]. Indole-acetic acid (IAA) production was tested according to Patten & Glick [24]. The ability of AK171 to grow at high temperatures (37 and 45 °C), and salt concentrations (0–5 M NaCl) was assessed using Zobell marine (ZM) media.

### Biofilm formation assessment

Pure cultures of AK171 were grown overnight in ZM broth (Lennox L Broth Base, Invitrogen) at 30 °C without shaking for 16 h. Fresh cultures were prepared by inoculating 3 mL ZM media with 100 µL of the pre-culture and allowed to grow for 4 h until reaching the exponential phase. A total of 20 µl of the exponential culture was spotted on Congo red (CR) plates (1% tryptone, 1% agar, 20 µg/mL Congo red, and 10 µg/mL Coomassie brilliant blue G250) [23]. Plates were incubated at 30 °C for up to 7 days to evaluate colony morphology and color. The quantitative assessment was done using the microtiter plate method (MTP) by crystal violet, as described previously [25]. 200 µl of ZM media was inoculated in wells of sterile 96-well microtiter polystyrene tissue culture plates. After cultivation for 24 h at 28 °C, the well contents were discarded and washed 3x with 200 µl sterile phosphate-buffered saline PBS (pH 7.2). The attached cells were precipitated with 200 µl of Sodium acetate (2%) and incubated for 5 min, then washed once with ddH_2_O. The biofilm was stained by adding 200 µl of crystal violet (0.1%) for 30 min at room temperature. The stained biofilm was washed 3x with ddH_2_O before diluting in 33% acetic acid and then measured the OD at 630 nm. OD_630nm_ < 0.120 was considered absent or weak biofilm, OD_630nm_ 0.120–0.240 as moderate biofilm, and OD_630nm_ > 0.240 as a strong biofilm.

### Arabidopsis salt stress tolerance assays

*A. thaliana* Col-0 seeds were surface sterilized for 10 min in 70% ethanol (*v/v*) solution supplemented with 0.05% triton-X, then washed 4 times with 100% ethanol. The seeds were left to dry in aseptic conditions on sterilized filter paper until use. Seed colonization with bacteria and plant growth conditions was conducted as described previously [26]. For the inoculation procedure, 100 µL of the bacterial culture (10E8 cells) mixed with 50 mL of the half-strength Murashige and Skoog basal medium (MS) (Sigma-Aldrich, St. Louis, MO, USA) (pH 5.8, adjusted with 10 mM KOH) and solidified with 0.9% Agar (Sigma-Aldrich). After that, five days-old seedlings with root lengths ~ 1–1.5 cm (6 seedlings/plate) were aseptically transferred using sterile toothpicks to the normal and stress plates. The first was the Standard plate Method (SPM), in which total agar medium in 90 mm square plates was used either filled with ½ MS as normal conditions or ½ MS stratified with 100 mM NaCl (5.844 g/L) as salt stress conditions. In the control (MOCK) with non-inoculated seeds, 100 µl SLB liquid medium was mixed with ½ MS medium. A Waterlogging Method (WM) was used to mimic the hypoxic stress conditions and salt stress conditions naturally occurring in the mangrove ecosystem. In this method, five-day-old seedlings were transferred on the top of ½ MS agar blocks without salt in normal conditions or amended with 100 mM NaCl in salinity conditions. The discs were also submerged in ½ MS liquid media supplemented with the same salt concentration, either none in normal (0 mM NaCl) or salinity conditions (100 mM NaCl). The plates of both methods were incubated in Percival with a light intensity of 150–200 µmol m^− 2^ s^− 1^ in 16 h light/8 h dark photoperiod at 22 °C for 15 days. Then, the total fresh weight (FW) was recorded to evaluate the effect of the bacterial treatment on the plant growth under both conditions.

### Biocontrol assessment against potential phytopathogens

The antagonistic effect of *T. mobilis* AK171 was assessed in vitro against a virulent strain of the plant pathogen *Pseudomonas syringae* pv. tomato DC3000 (*Pst*). In vitro testing was conducted by growing the tested bacteria (OD600nm = 0.2) on King’s B medium in an orbital shaker (120 rpm) at 28 °C. A mixture of 25 µL of this culture with 25 mL of cooled water yeast agar was prepared. After the agar solidified, 10 µL of AK171 (OD600nm = 0.2) was added as replicates of the antagonistic bacteria, with a spacing of 3.5 cm on a 9 mm petri dish. The plates were then incubated at 30 °C for 24–48 h, and the appearance of an inhibition zone was considered a positive result.

For the in vivo test, 100 µl of overnight culture of both the antagonistic bacteria (*T. mobilis* AK171) and the tested bacteria (Pst DC3000), were refreshed in 5 ml of their preferred media to reach the exponential phase. An amount of 50 mL of each bacterium was then adjusted to OD_600nm_ 0.2 and mixed with 50 mL ½ MS Agar. Then, surface sterilized seeds of *A. thaliana* Col-0 were sown on the solidified agar and transferred to Percival incubators within the same conditions of plant assay mentioned previously for 15 days. The survival rate was calculated compared to the negative control (survival value 20%) of Col-0 colonized with the tested bacteria alone (*Pst*, the pathogenic/tested strain). As a positive control, *Pseudomonas fluorescens* L111 was used as the antagonist strain (survival value > 40%). L111 was provided by Prof. David Dowling, Institute of Technology Carlow, Carlow, Ireland. We used Col-0 without colonization (MOCK) and Col-0 plants inoculated with AK171 alone as controls.

### Genomic DNA extraction

Total genomic DNA was extracted from a pure culture of AK171 using the GenElute™ Bacterial Genomic DNA Kit (Sigma Aldrich, Germany) according to the manufacturer’s instructions. DNA integrity, quality, and quantity were assessed by agarose gel electrophoresis 1%, NanoDrop 2000 spectrophotometer (Thermo Fisher Scientific, Schwerte, Germany), and the concentration by Qubit dsDNA high-sensitivity (HS) Kit (Thermo-Fischer Scientific).

### Whole genome sequencing and functional annotation

Genomic sequencing and assembly were performed at Novogene Bioinformatics Technology Co., Ltd. (Singapore). Single-molecule real-time (SMRT^®^) sequencing was performed on PacBio Sequel II/IIe system [27, 28]. FALCON software (falcon-kit = 1.8.1) was used for the whole genome assembly [29]. It follows the design of the previously developed Hierarchical Genome Assembly Process (HGAP), using greatly optimized components. Polishing and circularization of assembled genome done by Arrow (2.3.3) and circulator (1.5.5), respectively [30]. BUSCO (4.0.2) (Benchmarking Universal Single-Copy Orthologs, https://busco.ezlab.org) and CheckM quantitative measurements were used to assess genome assembly [31, 32]. Genome annotation was done for the coding gene, repetitive sequences, and non-coding RNA. For repeat annotation, the interspersed repetitive sequences were predicted using the RepeatMasker (http://www.repeatmasker.org/*)* [33]. The tandem Repeats were analyzed by the TRF (Tandem repeats finder) [34]. For ncRNA annotation, Transfer RNA (tRNA) genes were predicted by the tRNAscan-SE [34]. Ribosome RNA (rRNA) genes were analyzed using the RNAmmer [35]. BLAST predicted small nuclear RNAs (snRNA) against the Rfam database [36]. For coding gene prediction, Augustus (http://bioinf.uni-greifswald.de/augustus/*)* [37] and GeneWise (http://www.ebi.ac.uk/~birney/wise2/) software were used [38]. SMRT sequencing approach also detected methylated DNA bases. The MotifMaker detected and identified motifs associated with DNA modifications with default parameters. The functional annotation was performed using several databases such as respective GO (Gene Ontology), [39] KEGG (Kyoto Encyclopedia of Genes and Genomes) [40], KOG (EuKaryotic Orthologous Groups) [41], NR (Non-Redundant Protein Database) [42], Swiss-Prot, and TrEMBL [43]. Secondary metabolite-encoding gene clusters were identified using antiSMASH v.4.2.0 [44]. All the genomic features have been plotted using circalize R packages.

### Phylogenomic classification of AK171

For whole genome-based taxonomic analysis, genome sequence data were uploaded to the Type Strain Genome Server (TYGS) (https://tygs.dsmz.de*).* Two complementary ways were used to determine the closest type-strain genome. First, the AK171 genome was compared to genomes of all type strains available in the TYGS database via the MASH algorithm, a fast approximation of intergenomic relatedness, and the 10 type strains with the smallest MASH distances were chosen. Second, an additional set of 10 closely related type strains was determined via the 16 S rDNA gene sequences. These were extracted from the user genomes using RNAmmer [35]. Each sequence was subsequently BLASTed against the 16 S rDNA gene sequence of the currently 18,361-type strains available in the TYGS database. This was used as a proxy to find the 50 best-matching type strains (according to bitscore) for the AK171 strain genome and subsequently calculate precise distances using the Genome BLAST Distance Phylogeny (GBDP) approach under the “coverage” algorithm and distance formula d5 [35]. These distances were finally used to determine the 10 closest type strain genomes to the AK171. For the phylogenomic inference, all pairwise comparisons among the set of genomes were conducted using GBDP, and accurate intergenomic distances were inferred under the algorithm ‘trimming’, and distance formula d5 and 100 distance replicates were calculated each. Digital DDH values and confidence intervals were calculated using the recommended settings of the GGDC 3.0. The resulting intergenomic distances were used to infer a balanced minimum evolution tree with branch support via FASTME 2.1.6.1, including SPR postprocessing [45]. Branch support was inferred from 100 pseudo-bootstrap replicates each. The trees were rooted at the midpoint and visualized with PhyD3 [46]. Furthermore, Orthologous Average Nucleotide Identity Tool (OAT) software [47] calculated OrthoANI, ANIb, and ANIm values between the close strains of the AK171. Average Amino Acid Identity (AAI) was performed using the Java program EzAAI [48].

### Statistical analyses

The data from the plant screening assay were subjected to non-parametric one-way ANOVA or the Kruskal-Wallis test [49]. The statistical difference is based on the Paired t-test/ Dunn’s multiple comparisons tests. All statistical analysis was done using GraphPad Prism version 9.5.0 (525) software (https://graphpad.com).

### Data deposition

The genome sequence of *Tritonibacter mobilis* AK171 has been submitted to the NCBI GenBank database under accession number CP126134 in BioProject no. PRJNA973967.

## Results

### Morphological and plant growth promotion characteristics

**Fig. 1:**
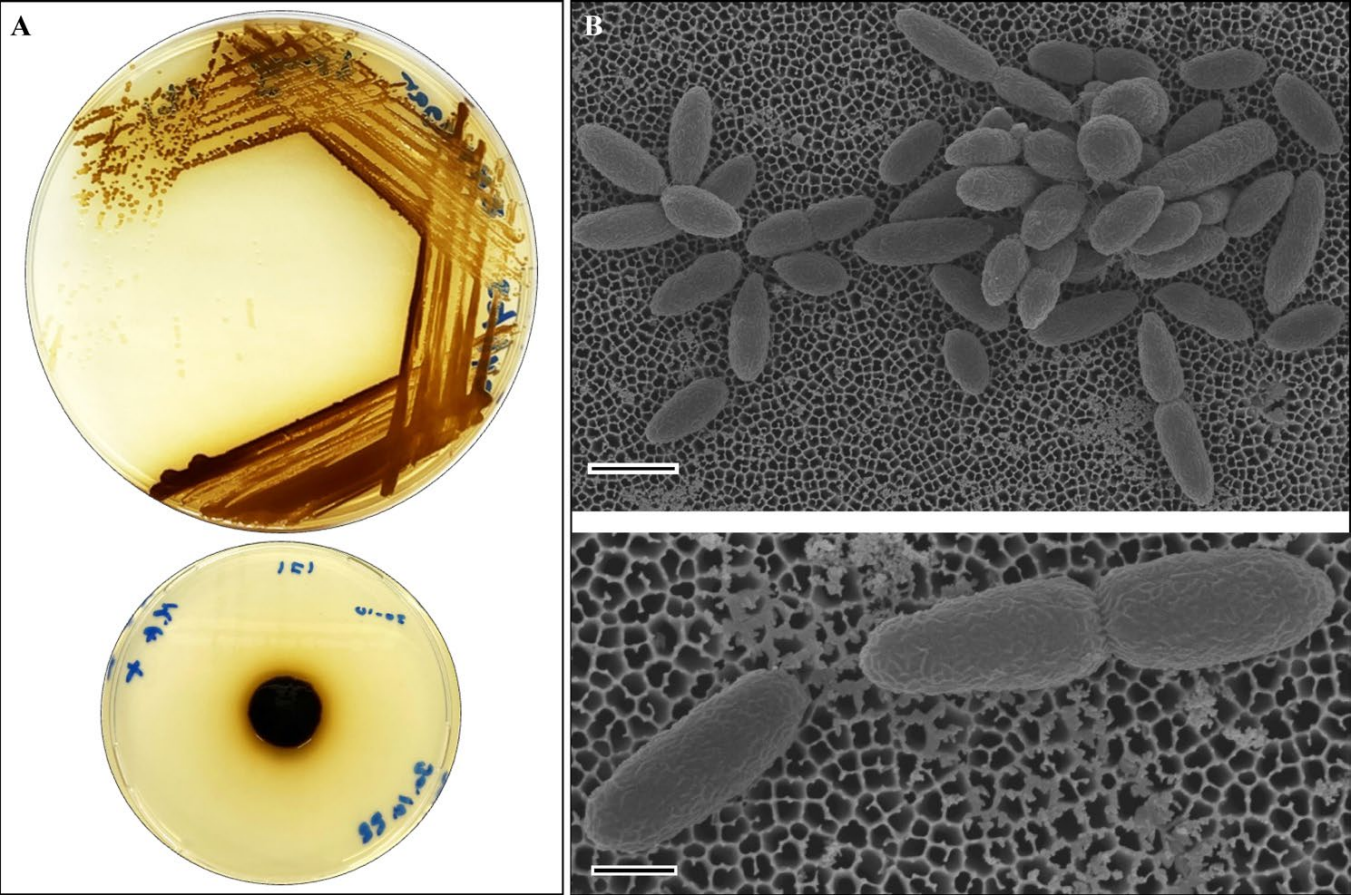
AK171 Morphology. (**A**) Colony morphology of *Tritonibacter mobilis* AK171 on ZM agar. (**B**) Scanning electron micrograph of T. *mobilis* AK171 growing in shaking (120 rpm) ZM broth for 3 h at 30 °C, showing rod shaped morphology (width 0.2 m and length 2.3 m). The cells aggregated in diplo-, star shape rosette, and few cells were elongated with different lengths ranging 3–5  m, Bar, 1 m

*Tritonibacter mobilis* AK171 colonies have a circular, cream-colored phenotype and measure 2 mm in diameter. The colonies turn brown after 24 h when growing on ZM agar at 30 °C. The cells were rod-shaped (length: 3–5 µM), forming a rosette arrangement as found by SEM (Fig. 1). AK171 can tolerate heat stress for up to 37 °C and grow well under saline conditions up to a concentration of 2 M NaCl (Supplement Table S1). The qualitative evaluation of PGP traits showed that AK171 could produce siderophores and IAA but could not solubilize phosphate nor produce hydrolytic enzymes (Supplement Table S1).

### Biofilm formation, plant growth promotion, and salinity stress alleviation

**Fig. 2:**
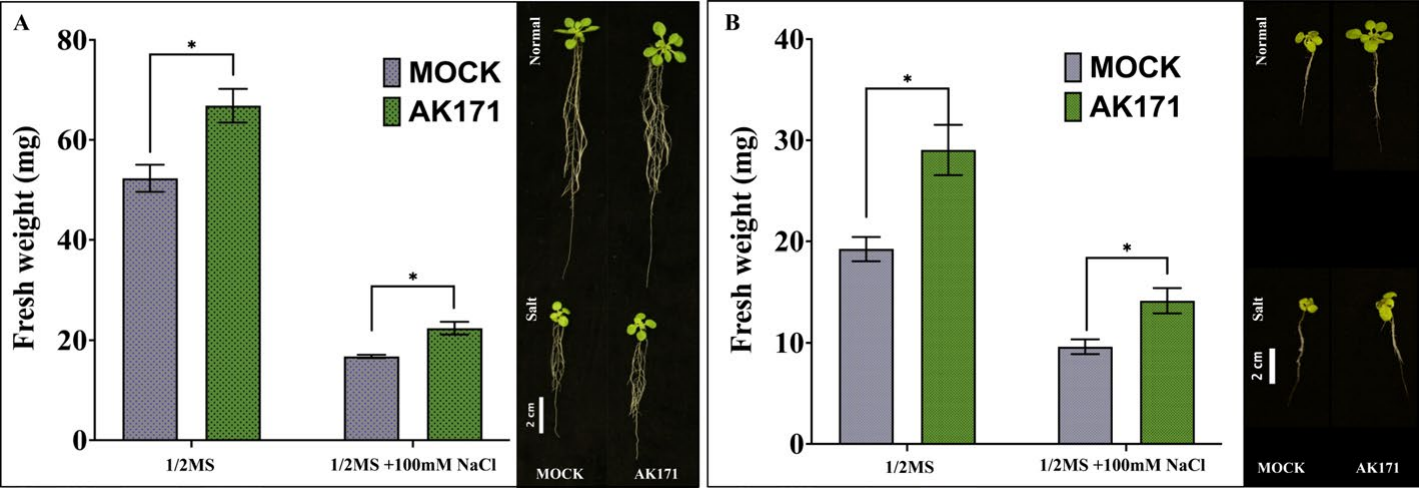
AK171 enhances salt stress tolerance of Arabidopsis. Fresh weight enhancement of *Arabidobsis thaliana* seedlings germinated with AK171 in 1/2MS and stress conditions 100mM NaCl compared to non-colonized plants (MOCK). It shows both the significance and the growth of 20-day-old seedlings. The seedlings were stressed in (**A**) a solid (aerated) phase and (**B**) in a submerged root environment. Bar, 2 cm

The qualitative biofilm test showed changes in the color of CR media, as shown in Supplement Figure S1-A, while the qualitative test resulted in OD_630nm_ = 0.24, indicating a moderate capacity to form biofilms. *In planta*, *T. mobilis* AK171 enhanced *A. thaliana* growth under both normal (½ MS) and salt stress conditions (½ MS + 100 mM NaCl). After 15 days of growth, *A. thaliana* seedlings treated with AK171 showed bigger shoot and root systems and an increment of > 25% in fresh weight compared with non-inoculated control plants (Fig. 2A) in both methods. Under the SPM method, AK171 increased the fresh weight of the seedling biomass of 20-day-old seedlings under normal and salinity-stressed conditions (Fig. 2B) by 27.8% and 33.6%, respectively. Using the WM method, the fresh weight measurements for 20-day-old seedlings exhibited similar increases under both normal and salinity-stressed conditions (35.2% and 47.3%, respectively) (Supplementary Table S2).

### Biocontrol potential of *Tritonibacter mobilis* AK171

In vitro experiments, it was observed that the growth of *Pseudomonas syringae* pv. tomato DC3000 (Pst) was inhibited, and a clear zone devoid of growth was visible around the *T. mobilis* AK171. This indicates that AK171 has the ability to suppress the growth of Pst in vitro. Furthermore, the results obtained from the in vivo assessment revealed a significant enhancement in the survival rate of *A. thaliana* when AK171 was introduced during the challenge with Pst. Particularly, the survival rate of *A. thaliana* increased by more than 50% when exposed to Pst in the presence of AK171, compared to the negative control, which employed only the pathogenic Pst strain. These findings provide compelling evidence that the incorporation of *T. mobilis* AK171 exerts a pronounced positive influence on the survival of *A. thaliana* in the presence of the pathogenic Pst strain (Supplementary Fig. 1-B).

### Genome features of *tritonibacter mobilis* AK171

**Fig. 3:**
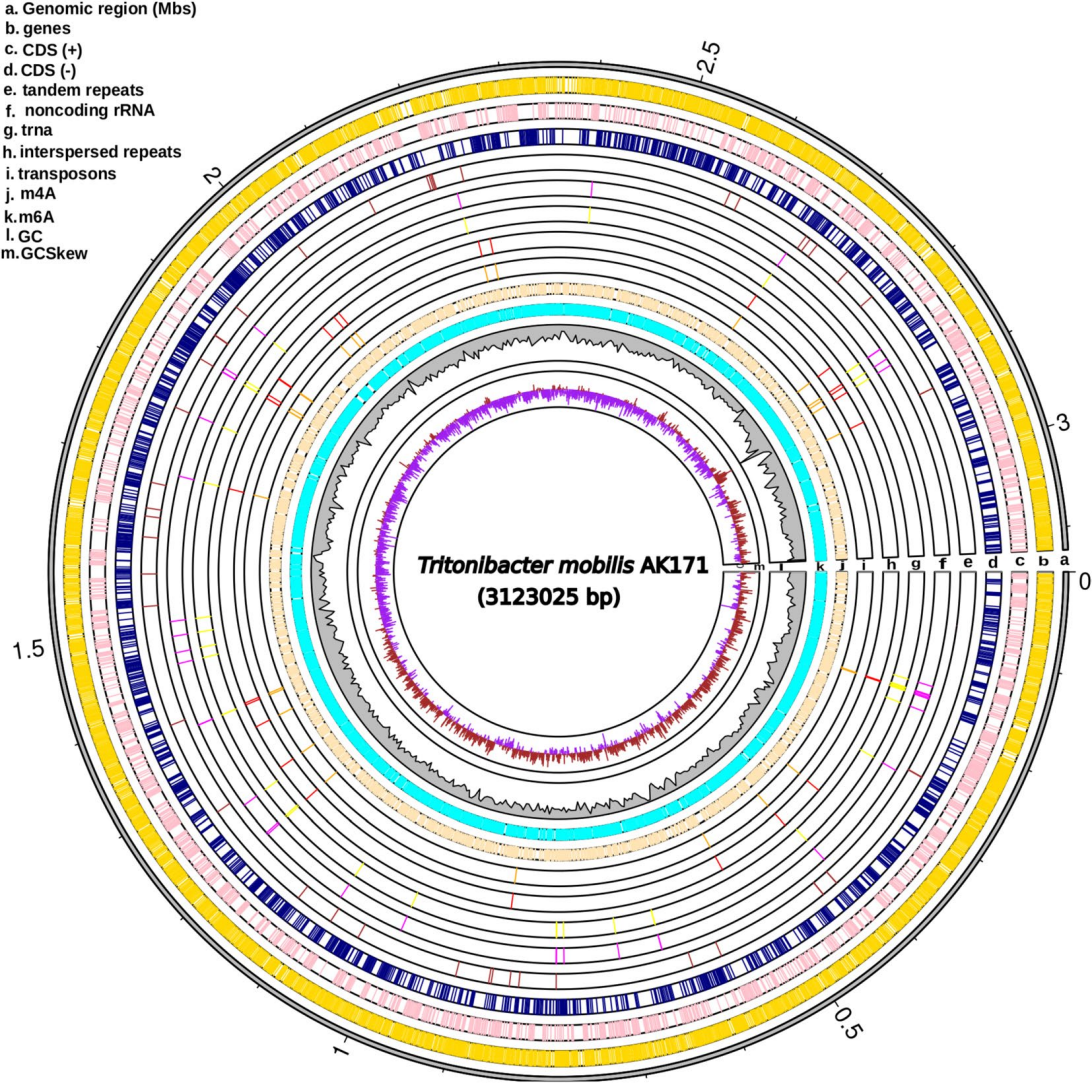
Genome features of AK171 genome. Circos map of AK171 genome. From the outer to the inner circle, representation is as follows: a. Whole genome sequences size split in Mbs (grey); b. genes (gold); c. forward strand coding sequences (pink); d. Reverse strand coding sequences (navy); e. Tandem repeats (brown); f. Noncoding rRNA (magenta); g. trna (yellow); h. Interspersed repeats (red); i. Transposons (orange); j. m6A (moccasin); k. m4A (cyan); l. GC content (grey)

Sequencing of *Tritonibacter mobilis* AK171 using PacBio technology resulted in 14,406 reads with a mean read length of 105,038 bp and estimated genome coverage of 484X (Table 1). The assembled genome consists of a circular contig of 3,123,025 bp with a GC content of 59.2%. The genome is assessed to be 92.7% complete, with zero contamination detected according to evaluations by BUSCO and CheckM, respectively. In total, 2980 coding genes have been annotated in AK171, covering 89.27% of the genome. RNA non-coding genes, including 39 for tRNAs, 3 for rRNAs, and 1 for sRNAs predicted in the genome (Fig. 3, Table [S1]). Interestingly, different types of repeats were also found in the genome, with a total of 29 interspersed and 98 tandem repeats. Modifications of about 2.75% and 3.46% of the genome were found for m6A (6-methyladenine) and m4C (4-methylcytosine), respectively. Motif string associated with m6A was detected as CRAGCAA (pos6), GANTC (pos2), GAGGNNNNNNGTC (pos2), GACNNNNNNCCTC (pos2), GACCTGG (pos2) and AV (pos1). From the 2980 genes, 2922 (98.1%) were classified into 2387 (80.1%) functional COG categories, KEGG 2868 genes (96.2%) GO 2083genes (69.9%), Pfam 2083genes (69.9%), and SwissPort 1184 genes (39.7%).

**Table 1:** Genome component of *Tritonibacter* sp. AK171

Genome size	3,123,025
Gene number	2980
Gene length	2,787,870
CDS	2980
% of Genome(Genes)	89.27
Gene average length:	936
tRNA	39
5s	1
16s	1
23s	1
Srna	1
LTR	17
DNA	2
LINE	6
SINE	1
RC	2
Unknown	1
TR	52
Minisatellite DNA	44
Microsatellite DNA	2
6-mA	2.75%
4-mC	3.46%
Genes assigned to:	
nr	2922
COG	2387
KEGG	2868
GO	2083
Pfam	2083
SwissProt	1184

By performing genome analysis using antiSMASH, AK171 was found in Figure S2 to harbor five gene clusters. These clusters include two gene clusters responsible for the biosynthesis of homoserine lactone and one gene cluster for beta lactone biosynthesis, exhibiting 13% similarity to corynecin I, II, and III. Additionally, AK171 possesses an ectoine biosynthetic gene cluster with 100% similarity to ectoine. Furthermore, there is a Type I polyketide synthase (PKS) biosynthetic gene cluster and an NPRS-like biosynthetic gene cluster, which do not exhibit similarity to any known clusters.

### Phylogenetic relationship analysis of AK171

**Fig. 4:**
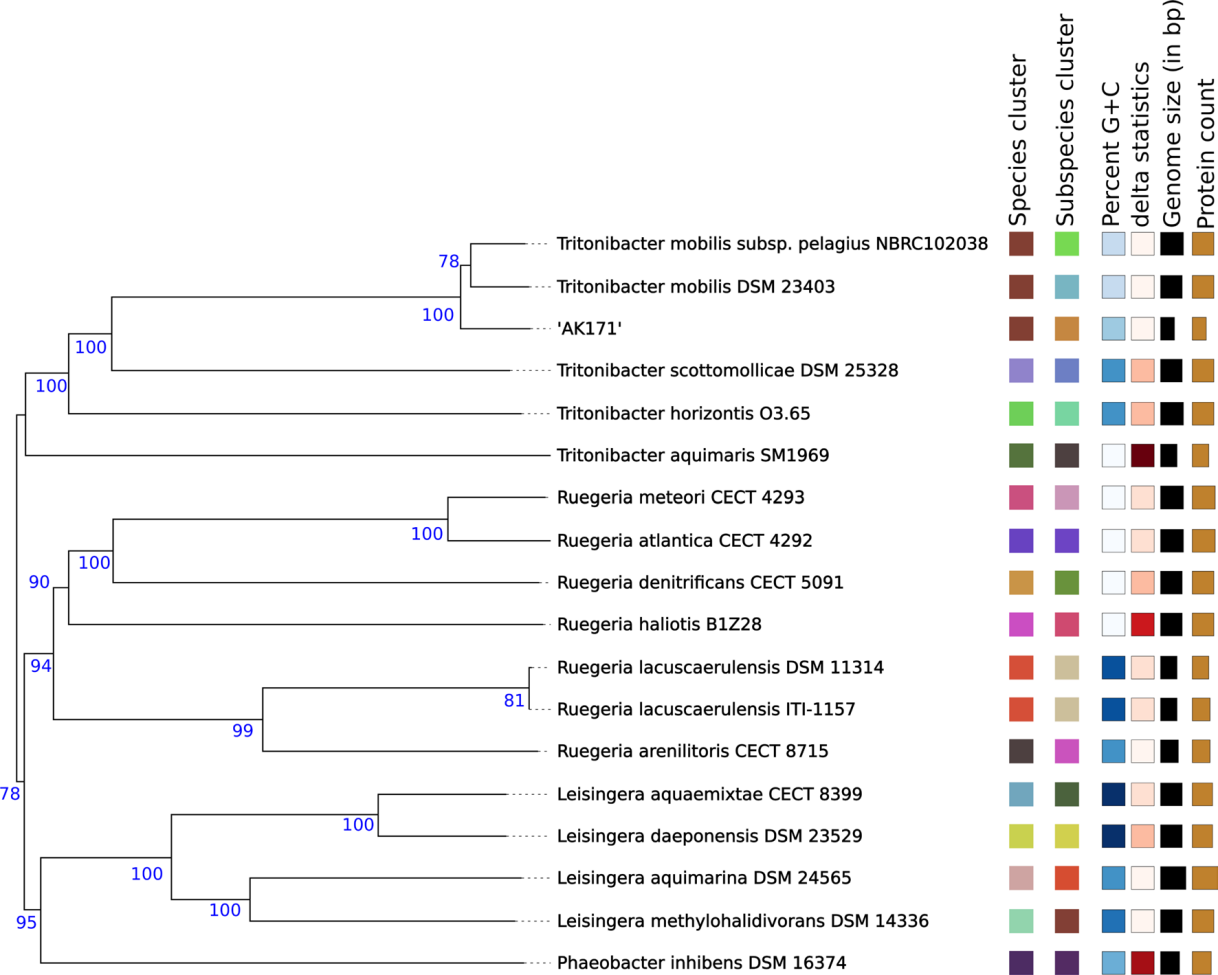
Phylogenomic classification of AK171 based on 16S. Tree based on 16S Basic Local Alignment Search Tool (BLAST) distance phylogenies (GBDP) using Type Strain Genome Server (TYGS) platform

**Fig. 5:**
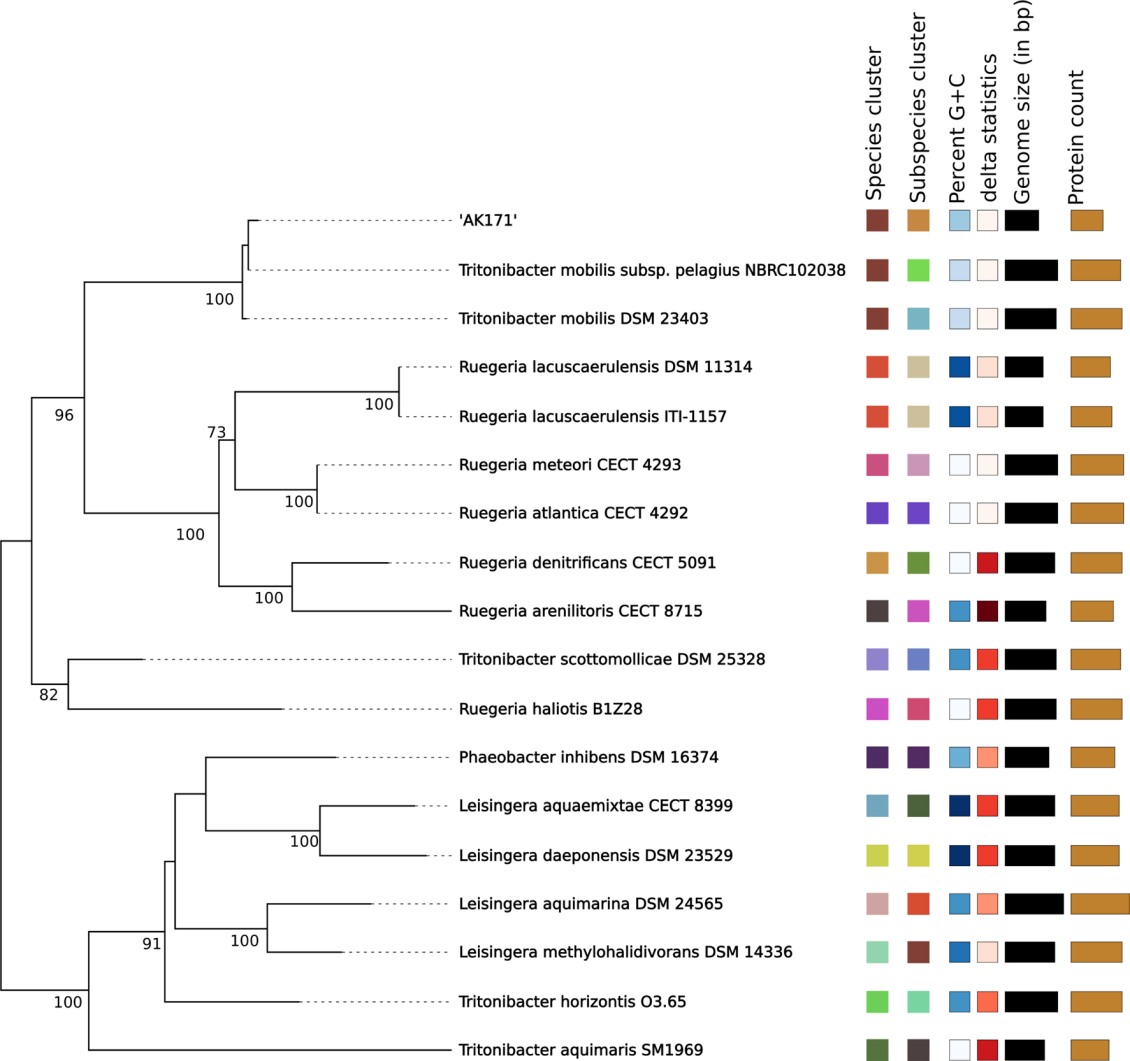
Phylogenomic classification of AK171 strain based on Whole Genome Sequence. Phylogenomic tree based on Genome Basic Local Alignment Search Tool (BLAST) distance phylogenies (GBDP) using Type Strain Genome Server (TYGS) platform

To determine the accurate taxonomic position of AK171, the 16 S (Fig. 4) and whole-genome-based taxonomic (Fig. 5) analysis was undertaken with the Type Strain Genome Server (TYGS) platform [50]. The TYGS results show that the AK171 strain is most closely related to *Tritonibacter mobilis* subsp. pelagius NBRC102038 (d0 = 60.6%, d4 = 73% and d6 = 64.2%) and *Tritonibacter mobilis* DSM 23,403 (d0 = 62.1%, d4 = 72.6% and d6 = 65.6%).

**Fig. 6:**
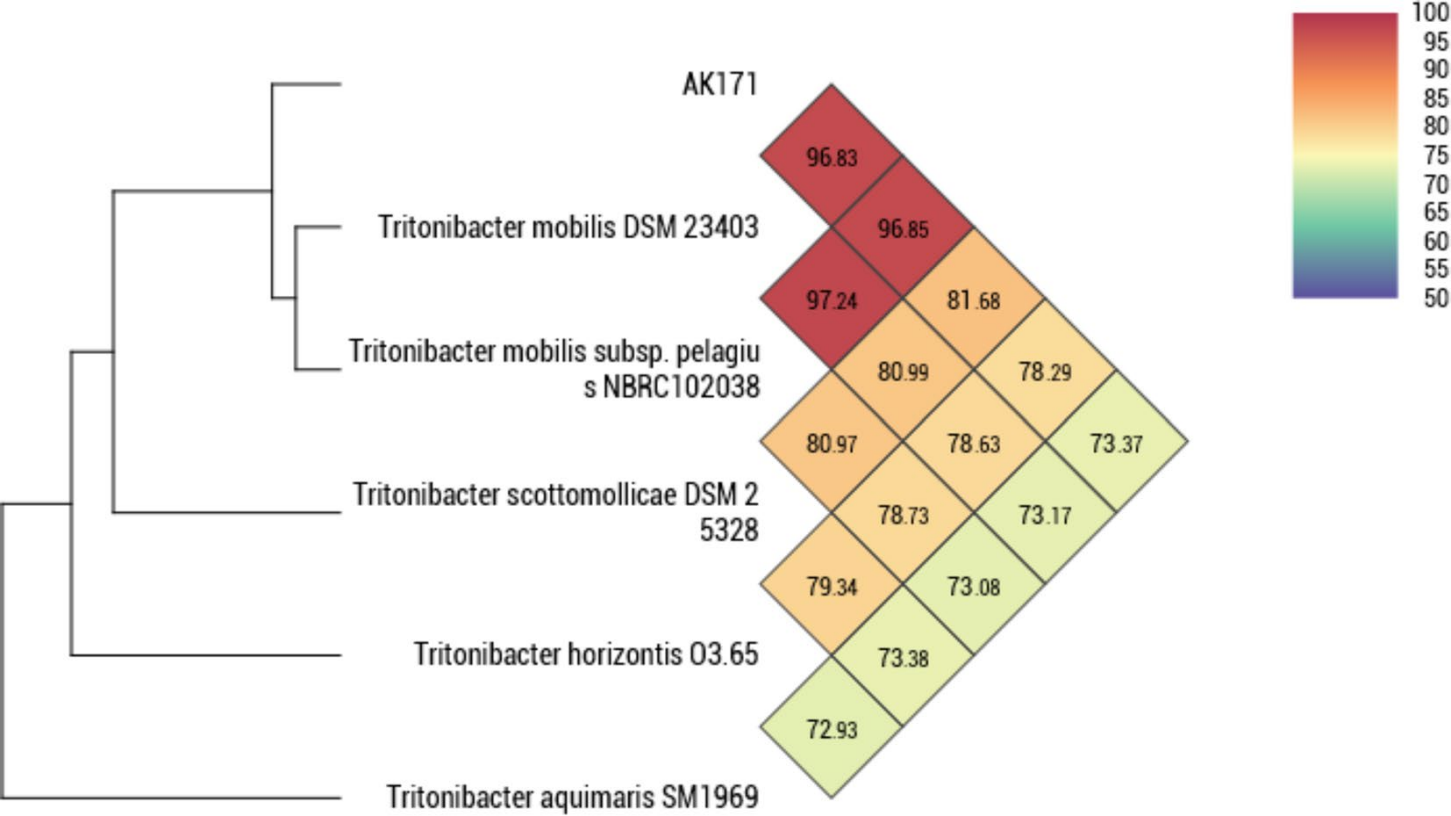
Phylogenomic classification of AK171 strain based on Average Nucleotide Identity Tool software (https://www.ezbiocloud.net/tools/orthoani*).* Heatmap presents OrthoANI values of AK171 strain and five closely related species. The color gradient represent its percentage identity

**Fig. 7:**
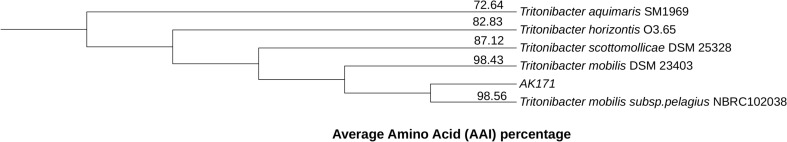
Phylogenomic classification of AK171 strain based on Average Amino Acid Identity (AAI). The top five highly identical bacterial species were analyzed for AAI

However, to check the reliability of evolutionary distance assessment between bacterial species based on digital whole genome comparison, average nucleotide identity (OANI, ANIb, and ANIm) was also measured. Five complete genomes of closely related AK171 genome were retrieved from the NCBI GenBank database and Orthologous Average Nucleotide Identity Tool (OAT) software. Their relationships and evolutionary distance were assessed based on ANI values. As shown in (Fig. 6, Table S3B, C and D) OrthoANI, ANIb, ANIm have a value of > 95% obtained with *Tritonibacter mobilis*. Identity at the level of amino acid also shows a high AAI percentage of > 98% with *Tritonibacter mobilis* (Supplementary Table S3E, Fig. 7).

## Discussion

### Adaptation of AK171 to stress conditions

The adaptation strategy of bacteria to salt stress conditions is multifaceted, involving the regulation of osmotic pressure, modifications in cell membrane composition, ion transport mechanisms, and the activation of stress response pathways. These adaptive mechanisms enable bacteria to survive and thrive in salt-rich environments by maintaining cellular homeostasis and protecting essential cellular components from the detrimental effects of high salt concentrations [51]. The growth of AK171 in highly saline media indicates that AK171 is a moderately halophilic bacterium that can tolerate NaCl concentrations up to 2 M (Supplementary Table S1). This indicates the ability to tolerate salt stress contributes to the survival of AK171 in the coastal intertidal plant environment of mangroves (*A. marina*) and the Red Sea. One primary mechanism through which bacteria adapt to high salt conditions is by regulating their internal osmotic pressure. They do this by accumulating or synthesizing compatible solutes, such as amino acids, sugars, and polyols, which help balance the osmotic pressure between the inside and outside of the cell. These compatible solutes act as osmoprotectants and prevent dehydration and shrinkage of the bacterial cells in hypertonic environments [52]. Genome mining of AK171 revealed the presence of genes related to the production of osmoprotectants, osmoregulation, and abiotic stress tolerance, such as heat, acidity, and oxidative stress (Supplementary Table S7). In addition, the superoxide dismutase (Sod2) gene (AK171_GM001633) can protect bacterial cells from oxidative stress, and two genes of KatG-producing catalase-peroxidase (AK171_GM002669). The gene clusters for cyclic lipopeptide (CLP) synthesis of *clpABPXSV*, including (AK171_GM000699, AK171_GM000143, AK171_GM001121, AK171_GM001122, AK171_GM002373, and AK171_GM002104) might also support plant growth with increased biomass and root architecture as found in a recent study on *A. thaliana* [53]. Several high-affinity K^+^ Transporter (HKT) gene TrKH/TrKG/ktrB classes of cation transporters (AK171_GM000029, AK171_GM000030) were also found, which addressed the salinity tolerance in bacteria and plants (Supplementary Table S7). Many studies directly elucidated that microorganisms accumulate polyhydroxyalkanoate (PHA) as an intracellular metabolic storage component, providing microorganisms with higher stress survival and strength [54]. A large number of polyhydroxyalkanoate (PHA) genes were found in the genome of the AK171 (AK171_GM001104, AK171_GM001105, AK171_GM001106, AK171_GM001107, AK171_GM001108, and AK171_GM001109).

Polyamines, such as putrescine, cadaverine, spermidine, and spermine, are also involved during oxidative, osmotic, heat, and salt stress [55, 56]. AK171 contains genes encoding polyamine transporters two copies of the *potABCD* (AK171_GM001814-17) and *potFGHI*: (AK171_GM002929-32), for the synthesis of putrescine from L-arginine (*speB*) AK171_GM001676, its conversion to spermidine AK171_GM001258 (*speE*), synthesis of spermidine from S-adenosyl-L-methionine AK171_GM001259 (*speD*) (Supplementary Table S5). The presence of genes for resistance to osmotic, oxidative, and salt stress suggests that AK171 possesses the potential to grow and tolerate these stresses, confirming the phenotypic assays (Supplementary Table S1).

Ectoine is a potential enzyme protector against stress as heat, cold, and high or low pH [57]. Several genes of action were found, including (Supplementary Table S7); ectA gene (AK171_GM002074) coding L-2,4-aminobutyric acid acetyltransferase, ectB gene (AK171_GM002073) coding for dat diamino butyrate-2-oxoglutarate transaminase and *ect*C gene (AK171_GM002072) coding for L-ectoine synthase were found. The presence of ectoine, an osmoprotectant compound, in the genome of AK171 might aid in its tolerance to salinity in its environment. In addition, ectoin-producing bacteria stimulate the root growth of treated seedlings under salinity stress [57].

Moreover, apart from synthesizing various osmoprotectants, carotenoid production has been identified as crucial for bacterial survival in the rhizosphere. Carotenoids also play a significant role in shielding bacteria against UV radiation and fostering the bacterial-plant interaction [58]. *Pantoea* sp. YR343 *Δcrt*B mutant, which lacks the phytoene synthase gene (*crtB*), served to underscore the significance of carotenoids in facilitating a successful plant-microbe interaction. As a result of the mutation, this strain was unable to produce carotenoids, suggesting the role of carotenoids in plant colonization. The genome mining revealed a large number of carotenoid biosynthetic genes this including (AK171_GM000050, AK171_GM000066, AK171_GM000257, AK171_GM000454, AK171_GM000487, AK171_GM000504, AK171_GM000541, AK171_GM000634, AK171_GM000882, AK171_GM000897, AK171_GM000954, AK171_GM001425, AK171_GM001509, AK171_GM001638, AK171_GM001720, AK171_GM002291, AK171_GM002306, AK171_GM002466, AK171_GM002651, and AK171_GM002928).

The ubiquitin (UBI) ubiEGH genes are involved in the terpenoid biosynthesis pathway and Volatile Organic Compounds (VOCs) antagonism [59]; in AK171, several genes, including (AK171_GM000214, AK171_GM002976, AK171_GM002851, AK171_GM000066, and AK171_GM000487) were found. A novel gene cluster (*moc*) associated with E1 catabolism was predicted in AK171 (Supplementary Table S7). The 3-hydroxybenzoate 4-monooxygenase (*moc*A; a single-component flavoprotein monooxygenase) identified in the moc cluster was found to be necessary, such as the gene *mocA* and of AK171 bacteria (AK171_GM001034). *moa*A (AK171_GM001881) and *moa*C for (cPMP synthase) (AK171_GM001466). These genes facilitated the biosynthesis of molybdenum cofactor (*moc*O) [60]. The adaptation strategies of AK171 to abiotic stress conditions are diverse and involve the production of stress proteins, modifications in cell membranes, osmoprotectant synthesis, pH homeostasis, and detoxification mechanisms. These strategies enable AK171 to survive and thrive in challenging environments by maintaining cellular homeostasis, reducing cellular damage, and preserving essential functions.

### AK171 is a potential PGPR for applications

Plant growth-promoting rhizobacteria hold great potential for various agricultural and environmental applications. Their unique capabilities stimulate plant growth and enhance plant health, making them valuable assets in sustainable agriculture and ecosystem management. One key application of PGPR is its ability to improve crop productivity. By enhancing nutrient availability and uptake, fixing atmospheric nitrogen, promoting root development, producing growth-promoting hormones, and protecting plants against pathogens and abiotic stresses. These beneficial effects contribute to increased crop yields, reduced reliance on chemical fertilizers, and improved soil fertility [61]. AK171 exhibited several plant growth-promoting traits involved in nutrient acquisition and modulating plant hormone levels that could possibly be responsible for growth promotion. The ability to produce IAA gives AK171 a potential application as PGPR. Several tryptophan biosynthesis pathway *trpCDEGS* genes were identified in AK171, including (AK171_GM001465, AK171_GM001463, AK171_GM001460, AK171_GM001462, AK171_GM000409, and *aro*CEF genes including (AK171_GM002695, AK171_GM002805, and AK171_GM000228) they are involved in synthesis and transporter of IAA (Supplementary Table S7). IAA is the most common auxin, which is crucial in developing the embryonic pattern, vascular tissues, leaf, secondary roots, flower initiation, and stem cell maintenance [62]. IAA was found to improve the development of rice deep root systems under stress conditions [63].

Bacteria use various mechanisms to compete for iron, an essential element that can limit their growth. These include specific iron uptake transporters, the secretion of diverse siderophores, and the synthesis of siderophore receptors to utilize siderophores from other microorganisms. A strong iron uptake system also helps protect host plants from pathogens by limiting the iron available to the siderophores [64]. AK171 genome mining revealed around 29 genes involved in iron- and heme-uptake. Among them, several iron ABC transporters: *Afu*ABC (AK171_GM001781, AK171_GM001782, and AK171_GM001783), as well as six genes coding for the iron complex-outer membrane receptor, MFS transporter and several ferrous iron uptake transporters system and 4 genes for heme exporter *ccmABCD* (AK171_GM001605, AK171_GM001606, AK171_GM001607, and AK171_GM001608) (Supplementary Table S5). The presence of efficient Fe-uptake systems can help AK171 compete for Fe in such environments.

Salicylate biosynthesis protein (AK171_GM002244) is involved in the biosynthesis of pyochelin, a siderophore molecule for iron acquisition [65]. As well as other siderophore genes including ABC-type Fe^3+^-siderophore transport system COG0609 (AK171_GM000303, AK171_GM001323, AK171_GM002562, and AK171_GM002887) and COG1120 ABC-type cobalamin/Fe^3+^-siderophores transport system (AK171_GM001324, AK171_GM002563, and AK171_GM002888) were also found in the genome of *T. mobilis* AK171 (Supplementary Table S5). This suggests that AK171 is not only able to solubilize Fe but can also import and export it to the host plant.

Bacterial production of VOCs like acetoin and 2,3-butanediol promotes plant growth [66]. AK171 is capable of producing acetoin and 2,3-butanediol 3-(methylthio)-1-propanol, tryptophol, this include *ilvABCDE* (AK171_GM002569, AK171_GM002830, AK171_GM002960, AK171_GM000846, AK171_GM000483, AK171_GM002323, AK171_GM002872, AK171_GM001743, AK171_GM001744, and AK171_GM001118) (Supplementary Table S7).

Many beneficial bacteria also produce a variety of antimicrobial compounds, thereby enhancing the plant’s resistance against pathogens. AK171 has the ability to produce an antibiotic agent called tropodithietic acid (TDA), which enhances microalgal health by killing pathogens [67]. Many genes of TDA biosynthesis were found in the AK171 genome (*paaK, paaH, hbd, fadB, mmgB, paaX, paaZ, paaG, paaI, paaG*) including AK171_GM000134, AK171_GM000153, AK171_GM000401, AK171_GM000402, AK171_GM000403, AK171_GM000404, AK171_GM000405, and AK171_GM001473 (supplementary Table S7). This could explain the biocontrol effect of *T. mobilis* AK171 towards DC3000.

In fact, *R.* mobilis was reported as a producer of TDA as a sulfate-containing compound with iron-chelating properties that may contribute to other functions, such as quorum sensing [18] and biofilm formation [68]. In particular, unlike siderophores, bioactive TDA is typically upregulated in the absence of iron. The weak iron-chelating properties of TDA indicate that iron sequestering is not its main function but is potentially related to its mode of action [69] or to symbiosis where the TDA–iron complex could serve as an iron reservoir [70]. This is also supported by a previous study of the Roseobacter group that metabolizes dimethylsulfoniopropionate (DMSP) [71]. In addition, TDA-producing *Tritonibacter* can catalyze DMSP and might produce dimethyl sulfoxide (DMS), which then serves as a source of sulfur or an anti-stress component that readily scavenges hydroxyl radicals and other reactive oxygen species [72].

Overall, the unique attributes and functions of AK171 make them valuable allies in sustainable agriculture, disease management, environmental remediation, and promoting plant health. Harnessing the potential of PGPR offers promising avenues for addressing agricultural and environmental challenges more efficiently, eco-friendly, and sustainably.

### AK171 chemotaxis, biofilm formation, and plant colonization

Many plant-associated bacteria are crucial in adhering to and colonizing plant roots. These bacteria produce several substances, including polysaccharides such as 3-deoxy-manno-octulosonate cytidyl (CMP), to facilitate this process. These contribute to the formation of biofilms, which serve as protective barriers and allow the bacteria to establish a stable presence on the root surface. The production of cellulose and exopolysaccharides by plant-associated bacteria is an important mechanism that enables them to interact with plants and establish beneficial relationships [73]. EAL (Glu-Ala-Leu) and GGDEF (Gly-Gly-Asp-Glu-Phe) domain proteins are common in bacteria and regulate biofilm formation. Diguanylate cyclases DGCs synthesize c-di-GMP, a key signaling molecule, while PDEs break it down. c-di-GMP controls the transition between biofilm and planktonic growth. High levels of c-di-GMP promote biofilm formation, while low levels favor planktonic growth. DGCs produce c-di-GMP by converting GTP, and PDEs degrade c-di-GMP into GMP. The balance between DGCs and PDEs is crucial for regulating bacterial growth lifestyles, especially in biofilm formation. [68]. In AK171, genome mining revealed the presence of 16 genes coding the EAL and GGDEF domain protein genes and diguanylate cyclases and phosphodiesterases. This includes AK171_GM000451, AK171_GM000620, and AK171_GM001571 coding GGDEF domain; AK171_GM000250, AK171_GM000555, and AK171_GM001049 coding EAL, and GGDEF domains; AK171_GM000964, and AK171_GM001576 coding c-di-GMP domain; AK171_GM000450, AK171_G000479, AK171_GM000555, AK171_GM000615, AK171_GM000620, AK171_GM001049, AK171_GM001229, and AK171_GM002786 coding diguanylate cyclases, and AK171_GM000677, AK171_GM000878, AK171_GM000964, AK171_GM000972, AK171_GM001048, AK171_GM001075, AK171_GM001098, AK171_GM001570, AK171_GM001576, AK171_GM001775, AK171_GM002311, AK171_GM002631, and AK171_GM002759 coding phosphodiesterases. In summary, in AK171, the EAL and GGDEF domain protein genes encode DGCs and PDEs, respectively, and their enzymatic activities are central players in regulating bacterial biofilm formation by modulating the levels of c-di-GMP. These mechanisms influence the behavior and characteristics of bacterial biofilms, including adhesion, colonization, matrix production, and motility.

In addition, the presence of type IV pili (T6P) in bacterial species has been closely associated with biofilm formation. These appendages aid in adhesion, provide structural support, enable interbacterial interactions, and facilitate bacterial colonization within the biofilm. AK171 has all genes essential for the formation, regulation, and assemble of the Type VI system, including AK171_GM002090, AK171_GM002091, AK171_GM002093, AK171_GM002097, AK171_GM002098, AK171_GM002099, AK171_GM002100, AK171_GM002103, and AK171_GM002104 (supplementary table S4). Bacterial biofilms can be regulated by a mechanism by which small signaling molecules called autoinducers are used for cellular communication, called quorum sensing (QS), allowing bacteria to regulate gene expression in a cell-density-dependent manner [74, 75]. The genome of AK171 contains genes for colonization, root surface adhesion, biofilm formation, quorum sensing, and secretion system (Supplementary Table S4). Understanding the role of T6P in biofilm development is crucial for deciphering the complex dynamics and behavior of bacterial biofilms, which can have significant implications in various fields, including medicine, industry, and environmental sciences.

In parallel, the marine Roseobacters representative genus, *Phaeobacter inhibens*, was studied in several aquaculture systems and is known for its potential to produce siderophores and acylated homoserine lactones (AHL). One gene (AK171_GM000963) was involved in N-acyl-L-homoserine lactone synthetase, and one gene (AK171_GM001294) coding Acyl-homoserine lactone (AHL) acylase was identified. The *rht*B genes encoding a homoserine/homoserine lactone efflux protein were found in the AK171 genome (AK171_GM000078) and *mlh*B, *chn*C genes encoding epsilon-lactone hydrolase (AK171_GM000417) (supplementary Table S7). Similar proteins were found in the antiSMASH results of the AK171 genome as homoserine lactones and beta lactones. These acylated homoserine lactones are pivotal in quorum sensing and antibacterial agents [76].

Several secretion systems prevent bacteria from being eliminated by the plant immune system [77]. The AK171 genome encodes type VI secretion system and several genes, including *secABDEFGY* (AK171_GM002861, AK171_GM002802, AK171_GM001611, AK171_GM000167, AK171_GM001610, AK171_GM000801, and AK171_GM000218). Another group of secretion proteins coded by *tatABC* (AK171_GM001001, AK171_GM001002, and AK171_GM001003) encodes sec-independent protein translocase protein. One gene encoding YidC/Oxa1 family membrane protein insertase was recovered (AK171_GM000249). Another gene (AK171_GM000833) *ftsY* encoding fused signal recognition particle receptor. The gene cluster impABCGHIJKLM encoding type VI secretion system consisting of genes was also recovered in the genome (AK171_GM002097, AK171_GM002098, and AK171_GM002099). Other genes encoding type VI secretion proteins: two copies of *vgrG* (AK171_GM002138 and AK171_GM002713), one copy of *hcp* (AK171_GM002100), *vasG* genes (AK171_GM002104), and *yajC* (AK171_GM001612) encodes the preprotein translocase subunit (Supplementary Table S4).

A number of polysaccharides biosynthesis genes were found in AK171: five polysaccharide biosynthesis proteins coded by *wza* gene (AK171_GM000661), *kpsS*, *lipB* capsular polysaccharide export protein (AK171_GM000662), COG2244 Membrane protein involved in the export of O-antigen and teichoic acid (AK171_GM002249), COG4221 NADP-dependent 3-hydroxy acid dehydrogenase (AK171_GM002496), COG4421 Capsular polysaccharide biosynthesis protein M (AK171_GM002782). Three gene clusters of lipopolysaccharide biosynthesis, lipopolysaccharide export system ATP-binding protein (lpt), including *lptABCF* (AK171_GM000024, AK171_GM000023, and AK171_GM000025), lipopolysaccharide export system permease protein *lpt*FG (AK171_GM001665, AK171_GM001666), LPS-assembly lipoprotein *lptE*, *rlpB* (AK171_GM000053), UDP-N-acetylglucosamine acyltransferase *lpx*ACD (AK171_GM001242, AK171_GM001917, and AK171_GM001394), *lpxB* lipid-A-disaccharide synthase (AK171_GM001244), *lpxK* tetraacyldisaccharide 4’-kinase (AK171_GM002501), *lpxL, htrB Kdo2*-lipid IVA lauroyltransferase (AK171_GM001597), and mannosyltransferase *lpcC* (AK171_GM001883). Two copies of *kdtA*, *waaA* 3-deoxy-D-manno-octulosonic-acid transferase (AK171_GM001882 and AK171_GM002500). *kdsA* 2-dehydro-3-deoxyphosphooctonate aldolase (KDO 8-P synthase) (AK171_GM000931 and AK171_GM001961) and *kdsB* 3-deoxy-manno-octulosonate cytidylyltransferase (CMP-KDO synthetase) (AK171_GM000014). One gene coding for the exopolysaccharide’s biosynthesis protein (AK171_GM002251). Overall, AK17a employs chemotaxis, biofilm formation, and plant colonization, which are important strategies to survive, adapt, and interact with their environment Plant/ Marin.

### AK171 central metabolism, ABC transporter, and two-component system

The genome of AK171 contains genes involved in the central carbon metabolism, including glycolysis (Embden–Meyerhof and Entner–Doudoroff pathways), pyruvate oxidation, tricarboxylic acid cycle, pentose phosphate pathway, and glyoxylate cycle. A number of genes are involved in the central carbon metabolism, including pyruvate phosphate dikinase (PPDK) (AK171_GM000477), triose-3-phosphate isomerase *tpiA* triose-P isomerase (TPI) (AK171_GM001839), ALDO fructose-bisphosphate aldolase (AK171_GM000797), Glyceraldehyde dehydrogenase (GAPDH) gene (AK171_GM000381, AK171_GM001493, AK171_GM002129, and AK171_GM002235), phosphoenolpyruvate carboxykinase pck (Pck) gene (AK171_GM002274) and *mae* genes (malate dehydrogenase), *glpX*-SEBP in gluconeogenesis (AK171_GM000624). Hence, these metabolic pathways should provide AK171 with the capacity to metabolize sugars and other carbon sources in the soil and plant root exudates.

Rhodobacterales (Roseobacter clade), including aerobic anoxygenic phototrophic (AAP) bacteria, represent an important part of marine microbial communities [78] and are thought to be important for the carbon cycle of the ocean by harvesting light by bacteriochlorophyll (BCl) and carotenoids. It could be investigated by looking for the photosynthetic gene cluster (PGC) in AK171. A key enzyme in this pathway, including the light-independent chlorophyllide reductase *bch*F (AK171_GM001606), the phosphorus metabolism AMGs [79] genes *pst*S (AK171_GM002863), and *pho*U (AK171_GM001354), and Proto-chlorophyllide reductase (AK171_GM000352). These proteins enable a broad view of the phylogeny of anoxygenic photosynthetic bacteria with a capacity to synthesize bacteriochlorophyll [80].

Phosphate transporter genes *pstAC* (AK171_GM001351 and AK171_GM001352) and potassium transporter genes (AK171_GM000029 and AK171_GM000030), *dhaS* (AK171_GM000109) gene coding for indol 3-acet-aldehyde dehydrogenase, and *trp*C (AK171_GM001465) coding for indole-3-glycerol-phosphate synthase.

Gene clusters of Cobalamin (Vitamin B12) *de novo* biosynthesis (Supplementary Table S7), *cobST, cobSV, cobPU*, and *cobABCDGKLMQW*, which is known to stimulate plant development were identified (AK171_GM001970, AK171_GM001972, AK171_GM002045, AK171_GM000421, AK171_GM002044, AK171_GM000562, AK171_GM000567, AK171_GM000568, AK171_GM000569, AK171_GM000571, AK171_GM000572, AK171_GM000574, AK171_GM000575, AK171_GM000576, AK171_GM000577, AK171_GM000578, AK171_GM000580, AK171_GM000785, AK171_GM001046, AK171_GM002424, and AK171_GM002425). Another two genes, btuB and btuR (AK171_GM001321 and AK171_GM000580), involved in salvage biosynthesis by absorbing corrinoids, were also identified. It was revealed that the Rhodobactreiales group might be considered probiotic microorganisms in aquaculture environments in maintaining the health of the culture system [81].

Other regulatory genes such as *ntrB, ntrC, ntrY*, and *ntrX* (AK171_GM001286, AK171_GM001287, AK171_GM001289, and AK171_GM001288) might be involved in nitrogen fixation and could allow this bacterium to grow in a nitrogen-deficient medium [2]. A two-component nitrogen fixation transcriptional regulator, *fix*ABSJ genes, together with *nif*H (AK171_GM000358, AK171_GM000766, AK171_GM002460, and AK171_GM002812) and *nif*U genes (AK171_GM001252). In addition, we identified a group of nitrogen utilization-related genes, *gln*B (AK171_GM001582 and AK171_GM002471) and *gln*G (AK171_GM001287) coding for nitrogen regulatory protein II, and *moe*A gene (AK171_GM001467) coding for molybdenum cofactor biosynthesis protein [82].

The F-type ATPase genes involved in the photosynthesis clusters were also found ATPF0A, ATPF0B, ATPF0C, ATPF1A, ATPF1B, ATPF1D, ATPF1G, ATPF1E (AK171_GM000702, AK171_GM000703, AK171_GM000704, AK171_GM000705, AK171_GM000706, AK171_GM002431, AK171_GM002432, AK171_GM002433, and AK171_GM002434). These genes are responsible for synthesizing ATP, which drives many processes in living cells [83]. A recent study showed that some F-type ATPase genes were upregulated by N-acetyl-5-methoxytryptamine (melatonin) treatment while downregulated by either salinity or melatonin plus salinity, which affected ATP synthesis and life processes, including photosynthesis, growth, and salinity stress response.

Bacterial dual lifestyle as free living in soil or association with plant roots provides access to various essential nutrients for bacterial proliferation and survival. Therefore, the genome of AK171 is equipped with a multitude of genes involved in the uptake, transport, and metabolism of nitrogen, sulfur, and carbon-based compounds. The list of metabolites and corresponding encoding genes, e.g., ABC transporters, Major Facilitator Superfamily (MFS) transporters, and Phosphotransferase systems (PTS), are listed in Supplement Table S5.

A large number of genes (72) encoding two-component systems (TCS) for rapid sensing and adjustment to changes in the external environment were present in AK171. The TCSs (two-component systems) in AK171 belong to several families, including OmpR, NtrC, NarL, and CheB/CheR. These TCSs play important roles in various cellular processes, e.g. in the phosphate starvation phoBR and phoPQ and the production of acid phosphatases. They regulate the cell’s response to phosphate limitation and aid in the production of acid phosphatases [84, 85]. Also in nitrogen metabolism, including *glnGL, glnKR*, and *narXL*. They play roles in the assimilation and utilization of nitrogen by the bacterial cell [3] the complete list of TCS is in supplementary Table S6. These mechanisms enable AK171 to find optimal growth conditions, enhance survival and resistance, acquire nutrients, establish beneficial relationships, and respond to environmental changes, ensuring their successful adaptation and colonization.

### AK171 bioactive secondary metabolites

Bioactive secondary metabolites produced by bacteria play a significant role in their adaptation and survival. These metabolites possess diverse biological activities, including antimicrobial, antifungal, and antiviral. One of the primary benefits of bioactive secondary metabolites for bacteria is competitive advantage. These compounds can inhibit the growth of other microorganisms, providing protection and resource competition. Bacteria can secure their niche in a specific environment by producing bioactive metabolites and fending off potential competitors [86]. The genome mining of AK171 reveals the presence of several operons of secondary metabolites; this includes Type I polyketide synthase (PKS) gene clusters such as T1PKS, terpene, and non-ribosomal peptide synthetases (NPRS-like) [87]. In bacteria the type 1PKS are novel and composed of modules that further consist of multiple domains covalently linked in a very long polypeptide to catalyze specific reactions, including the mini-PKS consisting of ketosynthase (KS), acetyltransferase (AT), and ACP and the accessory domains such as ketoreductase (KR), dehydratase (DH), enoyl reductase (ER), and methyltransferase [88]. T1PKS/nonribosomal peptide synthetase (NRPS) hybrid BGC was found in the clusters resulting from antiSMASH. Two *atoB* genes coding for acetyl-CoA C-acetyltransferase (AK171_GM000076 and AK171_GM001085). Two genes coding for cyclohexanone degradation (AK171_GM002241 and AK171_GM001085). One gene *murE* coding for UDP-N-acetylmuramoyl-L-alanyl-D-glutamate- 2,6-diaminopimelate ligase (AK171_GM000770). One gene *pat* coding for phosphinothricin acetyltransferase (AK171_GM000918). The PKS gene clusters have been distinguished as cis-acyltransferase (cis-AT) and trans-acyltransferase (trans-AT) based on the reliance of trans-AT PKS on separately encoded ATs for selecting α-carboxyacyl-CoA polyketide building blocks. Notably, the trans-AT PKS biosynthetic clusters are an evolving group of modular PKSs that are becoming more ubiquitously found in microbial genomes. In AK171, several genes encoding PKS synthesis, including *lpx*D coding for UDP-3-O-[3-hydroxymyristoyl] glucosamine N-acyltransferase (AK171_GM001394), *plsC* coding for 1-acyl-sn-glycerol-3-phosphate acyltransferase (AK171_GM001403), *pls*X coding for glycerol-3-phosphate acyltransferase (AK171_GM001700), *lpx*A coding for UDP-N-acetylglucosamine acyltransferase (AK171_GM001242), UDP-3-O-acylglucosamine N-acyltransferase (AK171_GM000918), *yaf*P coding acetyltransferase (AK171_GM002634), COG0204 coding for 1-acyl-sn-glycerol-3-phosphate acyltransferase (AK171_GM002267) and finally the *pps*E coding for phthiocerol/phenolphthiocerol synthesis polyketide synthase type I (Q7TXL6).

The Non-ribosomal Peptide Synthetases (NRPS) are large, multi-modular enzyme complexes involved in the biosynthesis of a diverse group of natural products known as non-ribosomal peptides (NRPs). NRPSs are found in bacteria, fungi, and plants and are crucial in producing bioactive compounds with various biological activities, including antimicrobial, anticancer, and immunosuppressive properties [87]. In AK171, several genes were found related to NRPS, including genes encoding saccharopine dehydrogenase (NAD^+^, L-lysine-forming) (AK171_GM000120) and type I glyceraldehyde-3-phosphate dehydrogenase (AK171_GM000381) (Supplement Figure S2). In addition, the AK171 genome encodes for several NRPS-like genes involved in lysine biosynthesis (Supplement Table S7). Lysine is an essential amino acid required for protein synthesis and is crucial in cellular metabolism. While lysine biosynthesis in bacteria is typically achieved through a series of enzymatic reactions. These NRPS-like genes are often called Lysine-activating peptide synthetase (LAP) genes. LAPs are similar in structure to NRPSs, with domains responsible for activating and incorporating amino acids. However, unlike traditional NRPSs, LAPs primarily function in lysine synthesis. NRPS-like LAP genes associated with lysine biosynthesis are found in various bacterial species. For example, the *dapX* gene in *Escherichia coli* [89] encodes a LAP involved in lysine synthesis. Another example is the *lysX* gene in *Bacillus subtilis* [90], which encodes a LAP involved in lysine activation. NRPS-like genes for lysine biosynthesis suggest the versatility of bacterial metabolic pathways and highlight the diversity of mechanisms for producing essential metabolites like lysine. These genes provide insights into the adaptive strategies AK171 employs to produce lysine and overcome limitations in the availability of exogenous lysine sources (Supplement Table S7).

Through the modular nature of NRPS assembly, the combination and arrangement of specific domains within the NRPS complexes can be altered to produce different peptide products. Due to their importance in synthesizing bioactive compounds, enzymes have become a valuable target for drug discovery and engineering novel peptide-based therapeutics [87, 91, 92], which gives AK171 a new avenue for industrial applications.

## Conclusions

The scarcity of freshwater resources and consequential yield losses pose significant challenges in modern agriculture. However, this study presents an innovative solution by utilizing *Tritonibacter mobilis* AK171, a halophilic marine bacterium capable of thriving in saline and waterlogged environments. The comprehensive genome sequence analysis of *T. mobilis* AK171 has shed light on the genetic mechanisms underlying its ability to adapt and thrive in salinity and waterlogging stress. Activating stress-responsive genes, producing specific enzymes and metabolites, and forming biofilms contribute to its remarkable tolerance to high salinity and waterlogging. Additionally, the presence of genes responsible for the synthesis of antimicrobial compounds, such as tropodithietic acid (TDA), unveils the potential for *T. mobilis* AK171 to effectively control phytopathogens, e.g., *Pseudomonas syringae* pv. tomato DC3000 is widely recognized for its ability to infect a diverse range of plant hosts, including various crops and ornamental plants. This antimicrobial activity further enhances its value for sustainable agriculture practices, creating eco-friendly alternatives to conventional chemical interventions. The findings from this study not only advance our understanding of plant-microbial interactions in saline and waterlogged environments but also provide promising opportunities for addressing the challenges of water scarcity and improving agricultural productivity in challenging conditions. By leveraging the genetic potential of *T. mobilis* AK171, sustainable and eco-friendly solutions can be realized, paving the way for a more resilient and efficient agricultural sector. Overall, this study highlights the enormous potential of halophilic marine bacteria and their ability to revolutionize agriculture in the face of water scarcity and environmental challenges.

### Electronic supplementary material

Below is the link to the electronic supplementary material.

Supplementary Material 1

Supplementary Material 2

## Data Availability

The genome sequence of Tritonibacter mobilis AK171 has been submitted to the NCBI GenBank database under accession number CP126134 in BioProject no. PRJNA973967.
