# Supplementary material for: Unraveling the genomic secrets of Tritonibacter mobilis AK171: a plant growth-promoting bacterium isolated from Avicennia marina
Source: BMC Genomics. 2024 Jul 5;25:672. doi: 10.1186/s12864-024-10555-0 (PMC11225332; doi:10.1186/s12864-024-10555-0)

**Supplement figures**

**Figure S1;** A-Biofilm formation on the Congo Red Agar using crystal blue (top) and brilliant blue (bottom) for a week at 30°C. B- The antagonistic test of the *Pseudomonas* DC3000 infectivity on *A. thaliana* seedlings (14 days old) inoculated with AK171 *T. mobilis*.


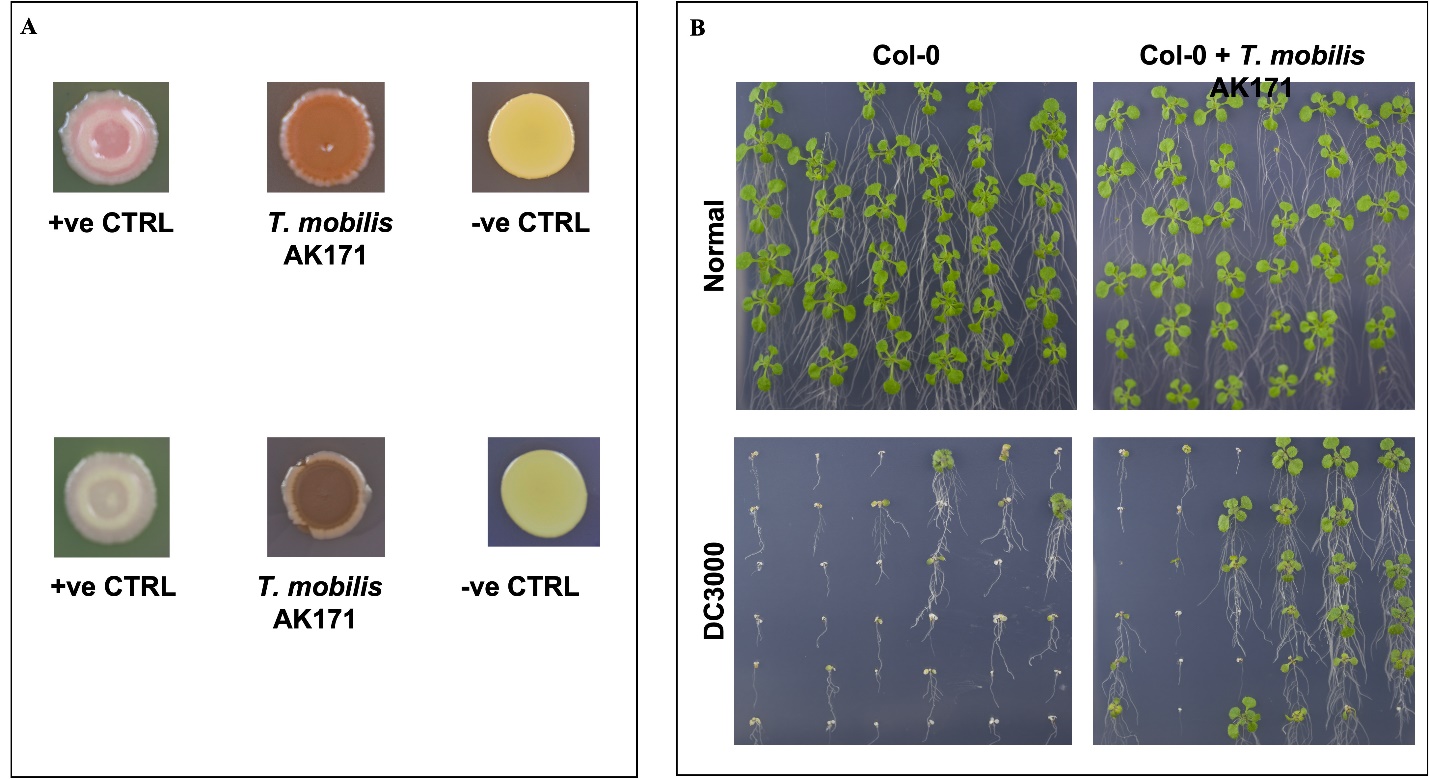


*+ve CTRL; *Pseudomonas* L111, -ve CTRL: *Isoptericola* sp.

**Figure S2:** Identification of secondary metabolites in AK171 using AntiSmash. Several regions were identified in AK171 encoding for HSL, ectoine and T1pKS and NRPS-like.


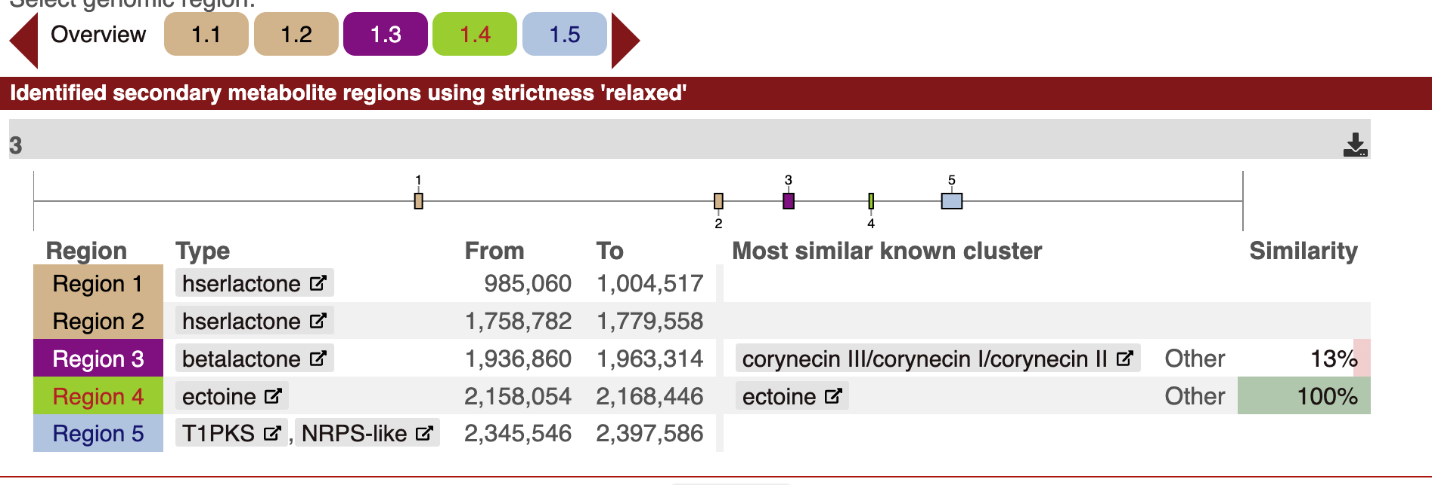

Supplement: Supplementary file 1 — Supplementary Material 1 [file 12864_2024_10555_MOESM1_ESM.docx]
